# Mechanisms Underlying Vasorelaxation Induced in Rat Aorta by Galetin 3,6-Dimethyl Ether, a Flavonoid from *Piptadenia stipulacea* (Benth.) Ducke

**DOI:** 10.3390/molecules191219678

**Published:** 2014-11-27

**Authors:** Cibério L. Macêdo, Luiz H. C. Vasconcelos, Ana C. de C. Correia, Italo R. R. Martins, Daysianne P. de Lira, Bárbara V. de O. Santos, Fabiana de A. Cavalcante, Bagnólia A. da Silva

**Affiliations:** 1 Programa de Pós-graduação em Produtos Naturais e Sintéticos Bioativos, Centro de Ciências da Saúde, Universidade Federal da Paraíba, João Pessoa, PB 58051-900, Brazil; E-Mails: ciberiolandim@hotmail.com (C.L.M.); henrique.luiz89@ltf.ufpb.br (L.H.C.V.); anacarolinacc@yahoo.com.br (A.C.C.C.); italo_rossi_@hotmail.com (I.R.R.M.); 2 Departamento de Farmácia, Faculdade Santa Maria (FSM), Cajazeiras, PB 58900-000, Brazil; E-Mail: daysianneplira@ltf.ufpb.br; 3 Departamento de Ciências Farmacêuticas, Centro de Ciências da Saúde, Universidade Federal da Paraíba, João Pessoa, PB 58051-970, Brazil; E-Mail: barbara@ltf.ufpb.br; 4 Departamento de Fisiologia e Patologia, Centro de Ciências da Saúde, Universidade Federal da Paraíba, João Pessoa, PB 58051-970, Brazil; E-Mail: fabianacavalcante@ltf.ufpb.br

**Keywords:** galetin 3,6-dimethyl ether, ion channels, calcium, phosphodiesterase, vasodilator

## Abstract

In this study, we investigated the relaxant action of galetin 3,6-dimethyl ether (FGAL) on rat aorta. The flavonoid relaxed both PMA‑ and phenylephrine (Phe)-induced contractions (pD_2_ = 5.36 ± 0.11 and 4.17 ± 0.10, respectively), suggesting the involvement of PKC and Phe pathways or α_1_ adrenergic receptor blockade. FGAL inhibited and rightward shifted Phe-induced cumulative concentration‑response curves, indicating a noncompetitive antagonism of α_1_ adrenergic receptors. The flavonoid was more potent in relaxing 30 mM KCl- than 80 mM KCl-induced contractions (pD_2_ = 5.50 ± 0.22 and 4.37 ± 0.12). The vasorelaxant potency of FGAL on Phe-induced contraction was reduced in the presence of 10 mM TEA^+^. Furthermore, in the presence of apamin, glibenclamide, BaCl_2_ or 4-AP, FGAL-induced relaxation was attenuated, indicating the participation of small conductance calcium-activated K^+^ channels (SK_Ca_), ATP-sensitive K^+^ channels (K_ATP_), inward rectifier K^+^ channels (K_ir_) and voltage-dependent K^+^ channels (K_V_), respectively. FGAL inhibited and rightward shifted CaCl_2_-induced cumulative concentration-response curves in both depolarizing medium (high K^+^) and in the presence of verapamil and phenylephrine, suggesting inhibition of Ca^2+^ influx through voltage-gated calcium channels (Ca_V_) and receptor operated channels (ROCs), respectively. Likewise, FGAL inhibited Phe-induced contractions in Ca^2+^-free medium, indicating inhibition of Ca^2+^ release from the sarcoplasmic reticulum (SR). FGAL potentiated the relaxant effect of aminophylline and sildenafil but not milrinone, suggesting the involvement of phosphodiesterase V (PDE V). Thus, the FGAL vasorelaxant mechanism involves noncompetitive antagonism of α_1_ adrenergic receptors, the non-selective opening of K^+^ channels, inhibition of Ca^2+^ influx through Ca_V_ or ROCs and the inhibition of intracellular Ca^2+^ release. Additionally, there is the involvement of cyclic nucleotide pathway, particularly through PDE V inhibition.

## 1. Introduction

Flavonoids are a large class of polyphenolic substances found in plants [1] known for their interesting activities in vascular diseases [2,3,4]. Several pharmacological effects have been described for this class of secondary metabolites, such as inhibition of enzymes involved in the synthesis of reactive oxygen species, such as xanthine oxidase, NADPH oxidase and lipoxygenase [5], increase in nitric oxide (NO) production in vascular smooth muscle [6] and spasmolytic activity in various models of smooth muscle [7,8]. Furthermore, some flavonoids are known for their actions on vascular tone, such as quercetin, kaempferol, luteolin, apigenin, catechin and epicatechin [1].

**Figure 1:**
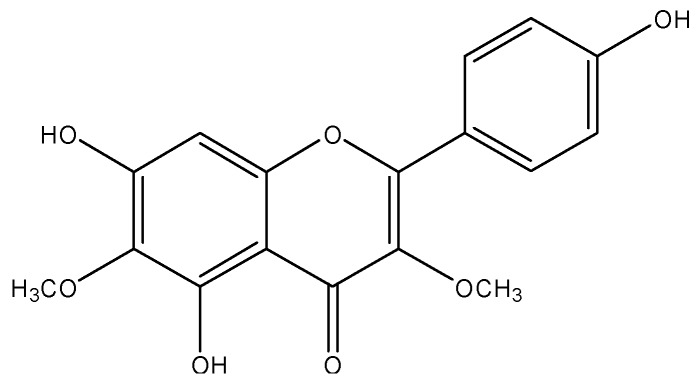
Chemical structure of galetin 3,6-dimethyl ether (FGAL).

Galetin 3,6-dimethyl ether (FGAL) (Figure 1), a flavonoid isolated from the plant *Piptadenia stipulacea* (Benth.) Ducke, has exhibited some pharmacological activities, such as antiviral [9], antinociceptive and anti-inflammatory activities in mice [2], as well as non-selective spasmolytic activity in smooth muscles (e.g., guinea-pig ileum and trachea and rat uterus and aorta). Moreover, this flavonoid has shown the highest relaxant potency in rat aorta, and this effect is independent of endothelium-derived relaxant factors (EDRF) [10].

Regarding the pharmacological effects described for flavonoids, these secondary metabolites appear to be candidates for the treatment of various diseases caused by disorders of smooth muscle, especially those affecting the cardiovascular system, such as hypertension, atherosclerosis and ischemic infarction [1]. Therefore, the aim of this work was to characterize the mechanisms involved in vasorelaxation induced by the flavonoid FGAL in rat aorta.

## 2. Results and Discussion

In blood vessels, the endothelium plays an important role in regulating vascular smooth muscle tone by releasing endothelium-derived relaxing factors (EDRF) [11], including endothelium‑derived hyperpolarizing factor (EDHF), nitric oxide (NO), prostacyclins and epoxyeicosatrienoic acids [12]. Despite this, it was previously demonstrated that the vasorelaxation induced by FGAL is independent of EDRF, since it relaxed aorta in both the absence and presence of endothelium in an equipotent manner [10]. On the other hand, several mechanisms are involved in endothelium-independent vasorelaxation, such as Ca^2+^ channel blockade, K^+^ channel opening, protein kinase C (PKC) inhibition, attenuation of Ca^2+^ release from the sarcoplasmic reticulum (SR) and phosphodiesterase (PDE) pathway inhibition [13]. Moreover, it has been demonstrated that flavonoids can produce vasorelaxation by different mechanisms, such as NO release from endothelium [14], PKC and PDE inhibition [15,16], blockade of Ca^2+^ influx through voltage‑sensitive Ca^2+^ channels (Ca_V_) [17] and K^+^ channel activation (IK_Ca_ and BK_Ca_) [18,19].

**Figure 2:**
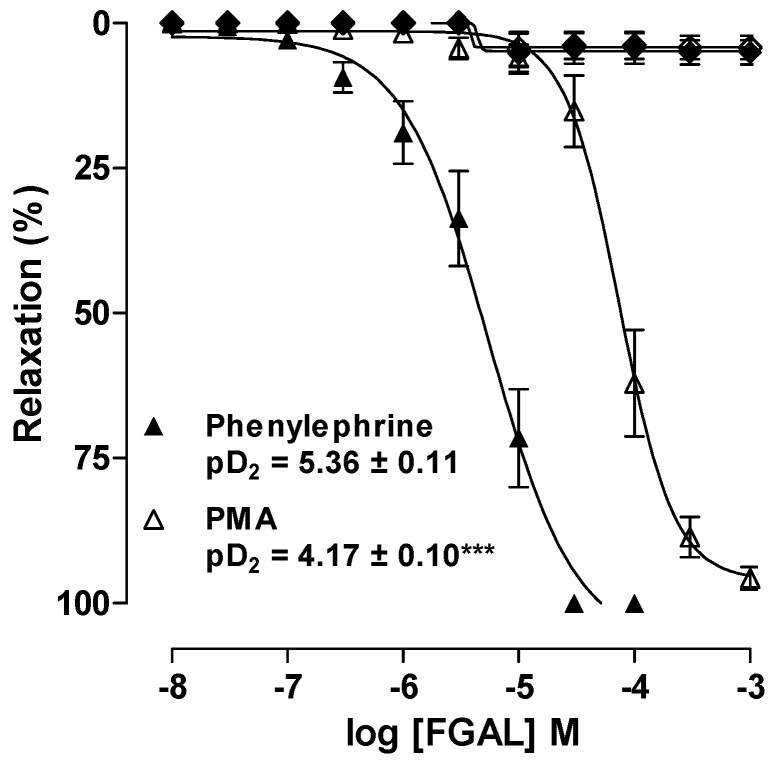
Effect of FGAL or vehicle on tonic contractions induced by 3 × 10^−7^ M Phe (FGAL: ▲, *n* = 5; vehicle: ♦, *n* = 3) or 10^−6^ M PMA (FGAL: Δ, *n* = 5; vehicle: ◊, *n* = 3) in rat aorta. Symbols and vertical bars represent the mean ± S.E.M., respectively. Student’s *t*-test, *** *p* < 0.001 (Phe* vs.* PMA).

PKC is a key protein to vascular smooth muscle contraction [20]. This protein kinase can both activate Ca_V_ and inhibit K^+^ channels, leading to Ca^2+^ influx and contributing to the contractile process. Phorbol esters, such as phorbol 12‑myristate 13-acetate (PMA), which are described as PKC stimulators, are used as exogenous activators of this protein kinase. Also, they have been known to induce sustained contraction in several arterial tissues [21,22]. FGAL (10^−8^ to 10^−3^ M) relaxed aorta pre‑contracted with 3 × 10^−7^ M phenylephrine (Phe) (pD_2_ = 5.36 ± 0.11) or 10^−6^ M PMA (pD_2_ = 4.17 ± 0.10). According to the pD_2_ values, FGAL was about 16-fold more potent in relaxing aorta pre‑contracted with Phe than with PMA. The vehicle did not show a significant relaxant effect in rat aorta pre‑contracted with either contractile agent (Figure 2).

The fact that PMA and Phe elicit contraction by different pathways suggests that the relaxant effect of FGAL in rat aorta involves both mechanisms. Therefore, the flavonoid can act on PKC (PMA exclusive target) as well as Ca_V_, K^+^ channels and Ca^2+^ mobilization from SR, which are involved in Phe signaling but are not directly affected by PMA [23]. Figure 2 shows that the inhibition of PKC is possibly involved, but that other targets (e.g., Ca_V_, K^+^ channels or others) are involved in a major manner. Moreover, we do not discard a possible direct antagonism of α_1_ adrenergic receptors by FGAL.

**Figure 3:**
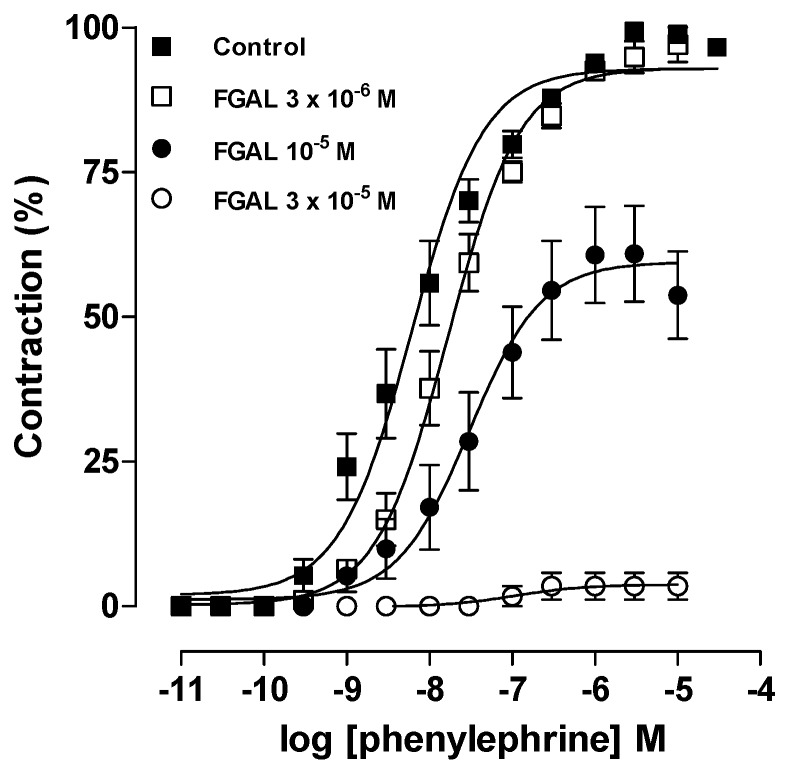
Cumulative concentration-response curves to phenylephrine in both the absence (■, control, *n* = 5) and presence of 3 × 10^−6^ (□, *n* = 3), 10^−5^ (●, *n* = 3) and 3 × 10^−5^ M (○, *n* = 3) of FGAL. Symbols and vertical bars represent the mean and S.E.M., respectively.

To assess the blockade of α_1_ adrenergic receptors by FGAL, the effect of the flavonoid on cumulative concentration‑response curves to phenylephrine (10^−11^ to 3 × 10^−5^ M) was investigated. FGAL (3 × 10^−6^ to 3 × 10^−5^ M) rightward shifted these curves in a non‑parallel manner with reduction in E_max_ and pD_2_ values of phenylephrine (Table 1, Figure 3). The FGAL pD_2_ value in inhibiting the effect of phenylephrine was 4.94 ± 0.04. In presence of a noncompetitive antagonist, the agonist has its maximum effect suppressed and abolished at higher concentrations of the antagonist [24]. Thus, according to the data obtained, FGAL showed the profile of a noncompetitive antagonist acting on α_1_ adrenergic receptors, which can be associated with a blockade of downstream pathways, such as Ca_V_, K^+^ channels and SR Ca^2+^ mobilization.

K^+^ channel activation and Ca_V_ blockade are two important mechanisms to promote vasorelaxation and are signaling pathway targets of α_1_ adrenergic receptor agonists, such as phenylephrine. Thus, we investigated whether these channels would be involved in the relaxant effect of FGAL. Accordingly, we evaluated the relaxation induced by FGAL in aorta pre-contracted with high (30 and 80 mM KCl) extracellular K^+^ concentration ([K^+^]_o_) [25]. By altering [K^+^]_o_, it is possible to determine if a drug has activity as a K^+^ channel opener or a Ca_V_ blocker, since K^+^ channel openers are more potent in relaxing a muscle pre-contracted with 30 mM than with 80 mM KCl, because this increase in [K^+^]_o_ to 80 mM prevents ion efflux, even in the case of the opening of K^+^ channels in the plasma membrane [26].

**Table 1:** E_max_ and pD_2_ values of phenylephrine in both the absence (control) and presence of FGAL (3 × 10^−6^ to 3 × 10^−5^ M) in rat aorta. Data are expressed as the mean ± S.E.M. (*n* = 3). One-way ANOVA followed by Bonferroni’s post-test: *** *p* < 0.001 (control* vs.* FGAL), ^##^ *p* < 0.01 (3 × 10^−6^* vs.* 10^−5^ M FGAL), **^¥¥¥^** *p* < 0.001 (10^−5^* vs.* 3 × 10^−5^ M FGAL). Nd = not determined.

[FGAL] M	E_max_ (%)	pD_2_
Control	100.0 ± 0.0	8.13 ± 0.18
3 × 10^−6^	94.9 ± 2.7	7.75 ± 0.10
10^−5^	60.9 ± 8.2 *** ^##^	7.49 ± 0.16
3 × 10^−5^	3.5 ± 2.3 *** ^¥¥¥^	Nd

**Figure 4:**
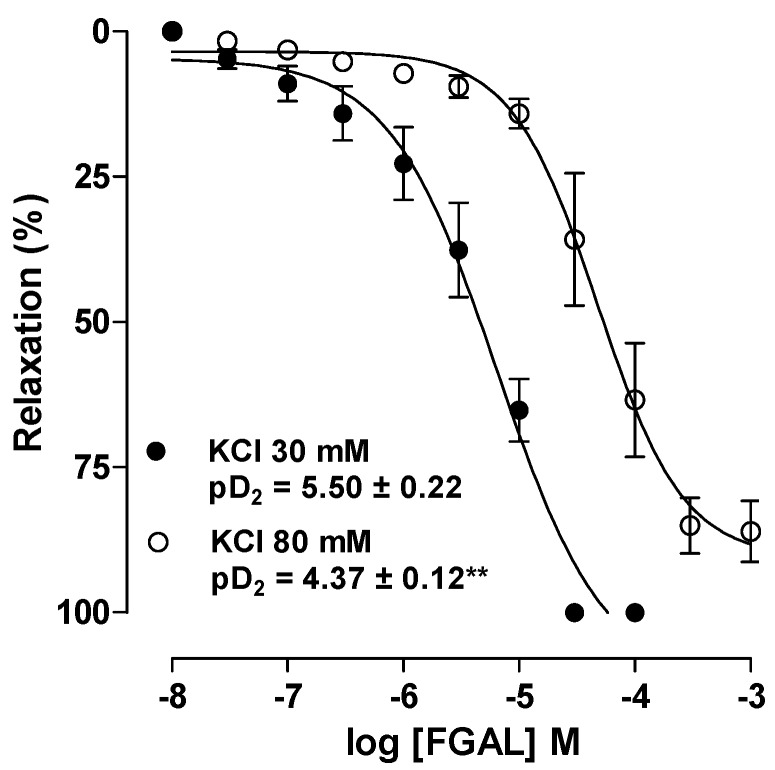
Effect of FGAL on tonic contractions induced by 30 mM KCl (●) or 80 mM KCl (○) in rat aorta. Symbols and vertical bars represent mean ± S.E.M., respectively. Student’s *t*-test, ** *p* < 0.01 (30 mM* vs.* 80 mM KCl).

FGAL (10^−8^ to 10^−3^ M) relaxed aorta pre-contracted with both 30 mM KCl (pD_2_ = 5.50 ± 0.22) and 80 mM (pD_2_ = 4.37 ± 0.12). According to the pD_2_ values, the flavonoid showed about 10-fold greater relaxant potency in rat aorta pre-contracted with 30 mM than with 80 mM KCl (Figure 4). This result indicates that FGAL activates K^+^ channels in relaxing rat aorta.

**Figure 5:**
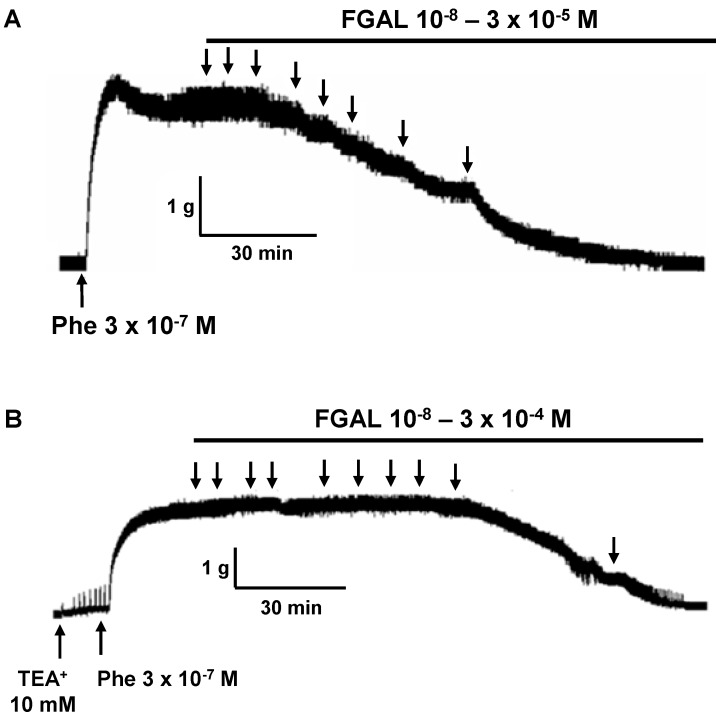
Representative records of relaxant effect of FGAL in rat aorta pre-contracted with 3 × 10^−^^7^ M Phe in both absence (**A**) and presence (**B**) of 10 mM TEA^+^. The arrows represent the addition of substances.

To confirm the activation of K^+^ channels by FGAL, the non‑selective blocker of these channels TEA^+^ (10 mM) was employed as a pharmacological tool. The relaxant potency of FGAL was reduced in the presence of the blocker (about 4-fold), confirming the participation of K^+^ channels in relaxation induced by FGAL (Figure 5, Table 2). The vascular smooth muscle expresses multiple K^+^ channel subtypes, where K_ATP_, K_ir_, K_V_, BK_Ca_ and SK_Ca_ are the most expressed subtypes [27]. Hence, the involvement of these subtypes of K^+^ channels was evaluated by employing their selective blockers. The relaxation induced by FGAL was not changed in the presence of 1 mM TEA^+^, excluding the participation of BK_Ca_. Conversely, the relaxant potency of the flavonoid was reduced in the presence of glibenclamide, BaCl_2_, 4-AP and apamin, indicating the activation of K_ATP_, K_ir_, K_V_ and SK_Ca_, respectively, by FGAL to induce vasorelaxation in rat aorta (Table 2).

**Table 2:** E_max_ (%) and pD_2_ values of FGAL in both the absence and presence of K^+^ channel blockers in rat aorta. Data are expressed as the mean ± S.E.M. (*n* = 5). One-way ANOVA followed by Dunnett’s post-test. * *p* < 0.05, ** *p* < 0.01 (FGAL* vs.* FGAL + blockers).

Compounds	E_max_ (%)	pD_2_
FGAL	100.0 ± 0.0	5.35 ± 0.11
10 mM TEA^+^ + FGAL	98.7 ± 1.3	4.71 ± 0.06 **
1 mM TEA^+^ + FGAL	92.8 ± 3.4	5.57 ± 0.17
10^−5^ M glibenclamide + FGAL	98.8 ± 1.2	4.79 ± 0.05 *
5 × 10^−8^ M apamin + FGAL	98.3 ± 1.1	4.75 ± 0.11 *
10^−4^ M BaCl_2_ + FGAL	89.1 ± 4.6 *	4.62 ± 0.17 **
10^−3^ M 4-AP + FGAL	86.2 ± 3.7 **	4.82 ± 0.11 *

Similar results are described in the literature for other flavonoids that promote relaxation in rat aorta through the activation of K^+^ channels; for example, pinocembrin activates K_ATP_, K_Ca_ and K_V_ [28], (‒)-epigallocatechin-3-gallate activates K_ATP_, SK_Ca_, IK_Ca_ and BK_Ca_ [29], and quercetin acts on BK_Ca_ [30].

Cytosolic calcium concentration ([Ca^2+^]_i_) increase in vascular smooth muscle cell is essential for its contraction and can occur by ion influx from the extracellular medium [31] or by release from intracellular stores [32]. The Ca^2+^ influx in vascular smooth muscle cells involves the opening of Ca_V_ and ROCs [33]. Pharmacological assays showed that the tonic contraction induced by high [K^+^]_o_ is mainly due to the depolarization of smooth muscle cells and consequent Ca^2+^ influx through Ca_V_, while the contraction induced by Phe is caused by Ca^2+^ influx through both Ca_V_ and ROCs [34,35,36]. Given this and since FGAL caused more potent relaxation (about 6-fold) in aorta pre-contracted with Phe (pD_2_ = 5.36 ± 0.11) than that with 80 mM KCl (pD_2_ = 4.37 ± 0.12), it is suggested that FGAL may reduce the Ca^2+^ influx through both Ca_V_ and ROCs.

**Figure 6:**
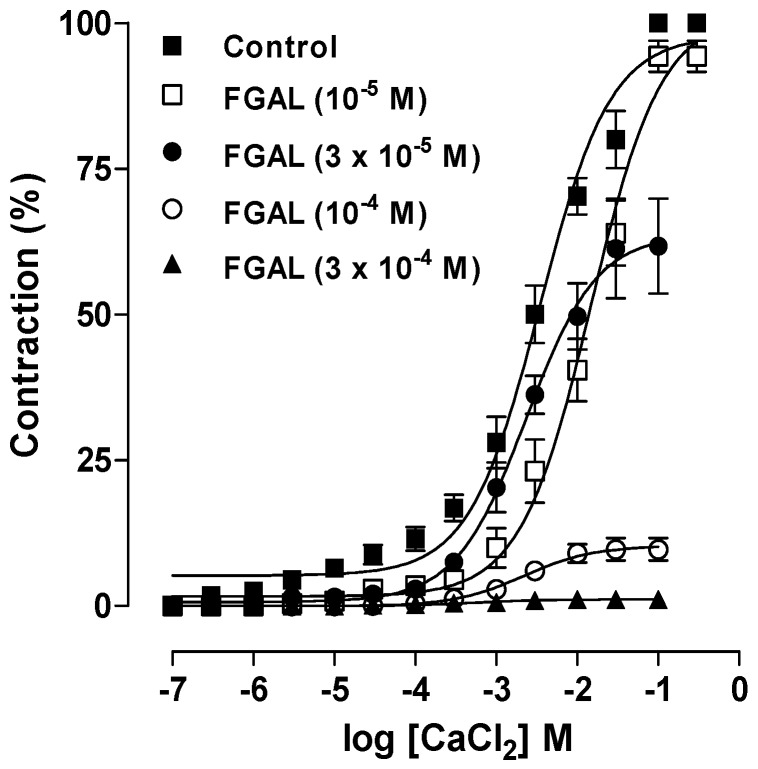
Cumulative concentration-response curves to CaCl_2_ in depolarizing medium (high K^+^) nominally without Ca^2+^ in both the absence (■, control, *n* = 5) and presence of 10^−5^ (□, *n* = 5), 3 × 10^−5^ (●, *n* = 5), 10^−4^ (○, *n* = 5) and 3 × 10^−4^ M (▲, *n* = 5) of FGAL. Symbols and vertical bars represent the mean and S.E.M., respectively.

To assess this hypothesis, we evaluated the effect of FGAL on CaCl_2_‑induced contractions in depolarizing medium nominally without Ca^2+^ to determine the inhibition of Ca_V_ by the flavonoid, and its effect on CaCl_2_‑induced cumulative concentration-response curves (10^−7^ to 10^−1^ M) in the presence of verapamil (Ca_V_ blocker) and Phe to determine the inhibition of ROCs. FGAL (10^−5^ to 3 × 10^−4^ M) inhibited these cumulative concentration‑response curvesand this effect was concentration dependent. These curves were rightward shifted in a non‑parallel manner with reduction in E_max_ and pD_2_ values of CaCl_2_ (Table 3, Figure 6). The pD_2_ value of FGAL in inhibiting the effect of CaCl_2_ was 4.44 ± 0.05.

**Table 3:** E_max_ and pD_2_ values of CaCl_2_ in both the absence (control) and presence of FGAL (10^−5^ to 3 × 10^−4^ M) in rat aorta. Data are expressed as the mean ± S.E.M. (*n* = 5). One‑way ANOVA followed by Bonferroni’s post-test: ** *p* < 0.01 and *** *p* < 0.001 (control* vs.* FGAL), ^###^ *p* < 0.001 (10^−5^* vs.* 3 × 10^−5^ M FGAL), ^¥¥¥^ *p* < 0.001 (3 × 10^−^^5^* vs.* 10^−^^4^ M FGAL). Nd = not determined.

[FGAL] M	E_max_ (%)	pD_2_
Control	100.0 ± 0.0	2.52 ± 0.10
10^−5^	94.3 ± 2.7	1.95 ± 0.12 **
3 × 10^−5^	61.7 ± 8.1 *** ^###^	2.69 ± 0.08
10^−4^	9.7 ± 1.9 *** ^¥¥¥^	Nd
3 × 10^−4^	1.1 ± 0.8 ***	Nd

**Figure 7:**
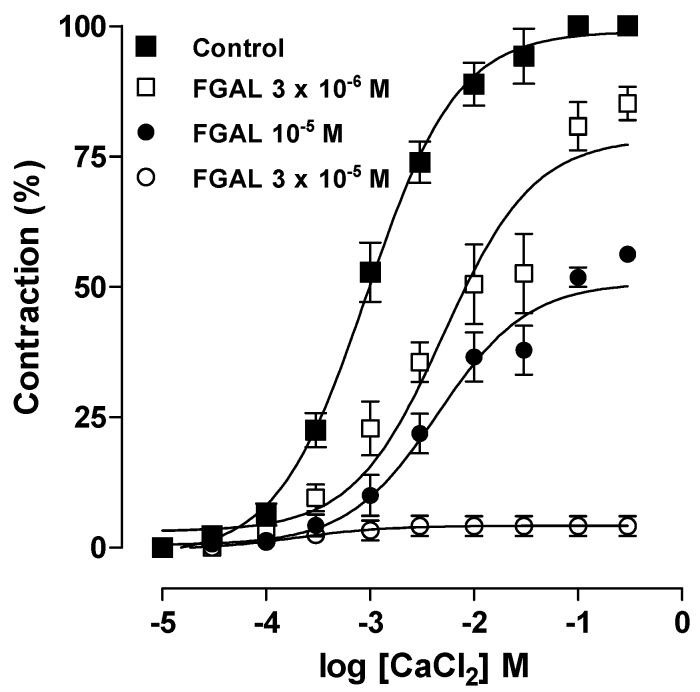
Cumulative concentration-response curves to CaCl_2_ in the presence of verapamil (10^−6^ M) and Phe (10^−6^ M), in both the absence (■, control, *n* = 5) and presence of 3 × 10^−6^ (□, *n* = 5), 10^−5^ (●, *n* = 5) and 3 × 10^−5^ M (○, *n* = 5) of FGAL. Symbols and vertical bars represent the mean and S.E.M., respectively.

In a similar way, FGAL (3 × 10^−6^ to 3 × 10^−5^ M) inhibited CaCl_2_-induced cumulative contractions (10^−5^ to 3 × 10^−1^ M) in the presence of verapamil and Phe (Table 4, Figure 7). The FGAL pD_2_ value in inhibiting the effect of CaCl_2_ was 5.07 ± 0.06. Together, these results confirm that FGAL inhibits Ca^2+^ influx by blocking both Ca_V_ and ROCs to relax vascular smooth muscle. However, comparing the inhibitory effect of FGAL in Figure 6 and Figure 7, the potency of the flavonoid was higher in the presence of verapamil and Phe than in their absence. This therefore indicates that FGAL acts mainly by inhibiting ROCs.

**Table 4:** E_max_ and pD_2_ values of CaCl_2_ in the presence of verapamil (10^−6^ M) and Phe (10^−6^ M), in both the absence (control) and presence of FGAL (3 × 10^−6^ to 3 × 10^−5^ M) in rat aorta. Data are expressed as the mean ± S.E.M. (*n* = 5). One-way ANOVA followed by Bonferroni’s post-test: * *p* < 0.05, ** *p* < 0.01 and *** *p* < 0.001 (control* vs.* FGAL), ^###^ *p* < 0.001 (3 × 10^−6^* vs.* 10^−5^ M FGAL), *p* ^¥¥¥^ < 0.001 (10^−5^* vs.* 3 × 10^−5^ M FGAL). Nd = not determined.

[FGAL] M	E_max_ (%)	pD_2_
Control	100.0 ± 0.0	2.97 ± 0.09
3 × 10^−6^	81.3 ± 4.6 **	2.28 ± 0.18 *
10^−5^	47.6 ± 4.6 *** ^###^	2.43 ± 0.20
3 × 10^−5^	4.1 ± 1.9 *** ^¥¥¥^	Nd

**Figure 8:**
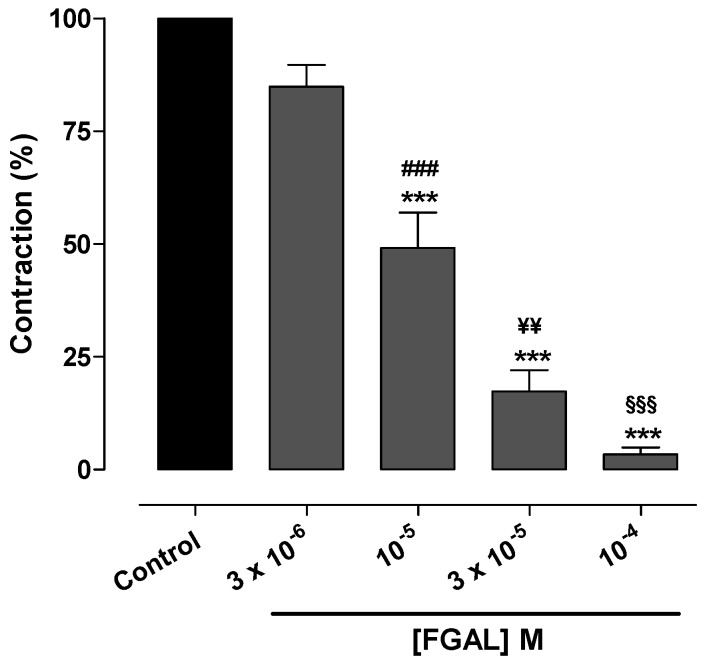
Effect of FGAL on contractions induced by 10^−6^ M Phe in Ca^2+^-free medium in rat aorta. One-way ANOVA followed by Bonferroni’s post-test, *** *p* < 0.001 (control* vs.* FGAL), ^###^ *p* < 0.001 (3 × 10^−6^* vs.* 10^−5^ M FGAL), *p* ^¥¥^ < 0.01 (10^−5^* vs.* 3 × 10^−5^ M FGAL), ^§§§^ *p* < 0.001 (3 × 10^−5^* vs.* 10^−4^ M FGAL), *n* = 5.

Increase in [Ca^2+^]_i_ may also occur due to Ca^2+^ release from intracellular stores, especially SR [37]; hence, in Ca^2+^-free medium, Phe-induced contraction occurs mainly due to Ca^2+^ release from SR. As can be seen in Figure 8, FGAL inhibited Phe-induced contractions in Ca^2+^-free medium in a concentration-dependent manner (E_max_ = 96.6% ± 1.5%; IC_50_ = 1.0 ± 0.1 × 10^−5^ M), supporting our hypothesis that the relaxant effect of FGAL involves the inhibition of Ca^2+^ release from SR.

PDEs are widely distributed in mammalian tissues and hydrolyze cAMP and cGMP, resulting in their inactive products 5'-AMP and 5'-GMP, which do not activate PKA and PKG, respectively, thus stopping the cell signaling mechanism dependent on increased cyclic nucleotides [38]. Substances able to raise intracellular levels of cAMP or cGMP show a strong relaxant effect, which can be due to PDE inhibition [39]. Some flavonoids from different plant species inhibit PDEs [40], for instance (‒)-epigallocatechina-3-gallate in rat aorta [29].

**Figure 9:**
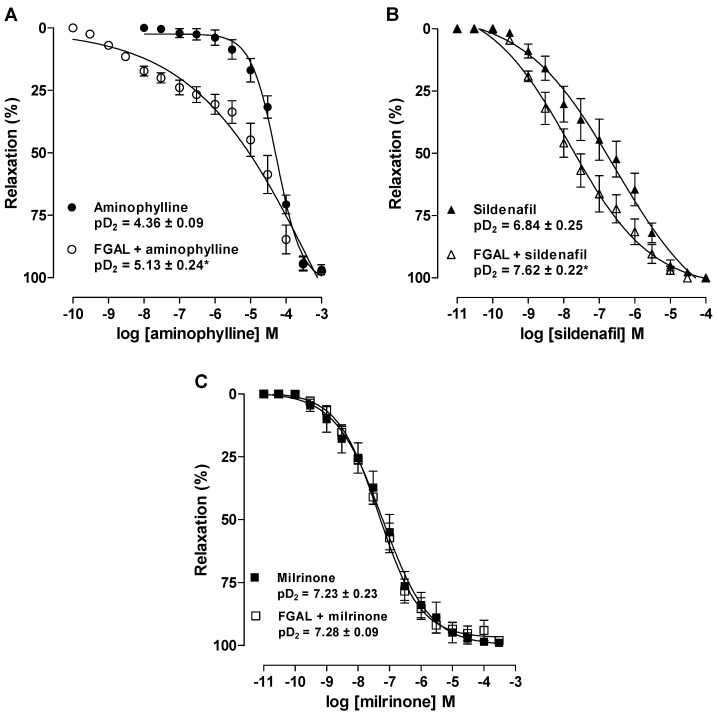
Effect of aminophylline (**A**, ●) sildenafil (**B**, **▲**) and milrinone (**C**, **■**) on tonic contractions induced by 3 × 10^−7^ M Phe in both the absence and presence of 3 × 10^−6^ M FGAL (○, ∆ and □, respectively). Symbols and vertical bars represent the mean and S.E.M., respectively. Student’s *t*-test, * *p* < 0.05 (aminophylline/sildenafil* vs.* FGAL + aminophylline/sildenafil), *n* = 5.

Therefore, to assess the possible involvement of cyclic nucleotide-PDE pathway, we determined the relaxant effect of aminophylline, a non-selective PDE inhibitor, in both the absence and presence of FGAL. The relaxant effect induced by aminophylline (10^−10^ to 10^−3^ M, positive control) (pD_2_ = 4.36 ± 0.09) was potentiated about 4-fold in the presence of FGAL (pD_2_ = 5.13 ± 0.24), confirming that FGAL inhibits cyclic nucleotide-PDE pathways to relax rat aorta. Additionally, in vascular smooth muscle, PDE III and V are the most expressed PDE subtypes [38], and interestingly, the relaxation curve induced by sildenafil (10^−8^ to 10^−4^ M) (pD_2_ = 6.84 ± 0.25), a PDE V inhibitor, was potentiated about 9-fold in the presence of FGAL (pD_2_ = 7.62 ± 0.22); however, the relaxation induced by milrinone (10^−8^ to 3 × 10^−4^ M) (pD_2_ = 7.23 ± 0.23), a PDE III inhibitor, was not changed in the presence of the flavonoid (pD_2_ = 7.28 ± 0.09) (Figure 9). 

Thus, these results indicate that PDE V but not PDE III pathway is involved in relaxation induced by FGAL in rat aorta.

## 3. Experimental

### 3.1. Chemicals

The flavonoid FGAL was obtained according to the reported method [2]. Aminophylline, apamin, barium chloride (BaCl_2_), Cremophor EL^®^, glibenclamide, phenylephrine (Phe), phorbol 12-myristate-13-acetate (PMA), tetraethylammonium chloride (TEA^+^), verapamil and 4-aminopyridine (4-AP) were obtained from Sigma-Aldrich (Duque de Caxias, RJ, Brazil). Milrinone was obtained from Sanofi-Aventis (São Paulo, SP, Brazil) and sildenafil was obtained from Nutrifarm (São Paulo, SP, Brazil). All substances were dissolved in distilled water, except glibenclamide, which was dissolved in ethanol and diluted in distilled water, and FGAL, which was dissolved in 3% Cremophor EL^®^ and diluted in distilled water to 10^−2^ M and further diluted according to concentration required for each experimental protocol. The final concentration of Cremophor EL^®^ in the organ bath never exceeded 0.3% (v/v), which was demonstrated to be devoid of significant observable effects on vascular muscle tone (Figure 2).

### 3.2. Animals

Male Wistar rats (*Rattus norvegicus*) weighing 250–350 g from Bioterium Professor Thomas George of Centro de Biotecnologia (CBiotec/UFPB) were used. The animals were maintained in a 12 h light/dark cycle (lights on: 06:00–18:00 h), under controlled temperature (21 ± 1 °C), with free access to food and water. Animal welfare and experimental procedures were followed in accordance with the Ethical Principles for Care and Use of Laboratory Animals of the Brazilian Society for Laboratory Animal Science, approved by the Animal Research Ethics Committee (CEPA) of “Laboratório de Tecnologia Farmacêutica” (LTF/UFPB, protocol 0105/10).

### 3.3. Preparation of Rat Aortic Rings

The animals were euthanized by cervical dislocation followed by sectioning of cervical vessels. Rat aorta was immediately removed, immersed in Krebs solutions and bubbled with carbogen mixture (95% O_2_ and 5% CO_2_). The Krebs solution composition was (mM): NaCl (118.0), KCl (4.55), MgSO_4_ (5.7), KH_2_PO_4_ (1.1), CaCl_2_ (2.52), NaHCO_3_ (25.0) and glucose (11.0), with pH adjusted to 7.4. The Krebs depolarizing solution nominally without Ca^2+^ was made with 80 mM KCl in equimolar exchange for NaCl, and the Ca^2+^‑free Krebs solution with the addition of 3 mM EDTA, both with omission of CaCl_2_. Aortic rings (2–3 mm) were immersed in organ baths with 5 mL of Krebs solution, at 37 °C and bubbled with carbogen mixture. 

To record isometric contractions, aortic segments were suspended with steel rods and connected to force transducers (FORT-10) attached to an amplifier (TMB4M), both from World Precision Instruments (Sarasota, FL, USA) and connected to an A/D converter into a PC running Biomed^®^ software (BioData, Ribeirão Preto, SP, Brazil). The resting time of aorta was 60 min in a preload tension of 1 g (baseline). During the organ resting phase, the solution was changed every 15 min to avoid metabolite accumulation. In the experiments, the integrity of endothelium was not verified, since the relaxant effect exhibited by FGAL was similar either in its absence and presence [10].

### 3.4. Experimental Protocols

#### 3.4.1. Effect of FGAL on Rat Aorta Pre-Contracted with Phe or PMA

After the initial procedures, a contraction was evoked with 3 × 10^−7^ M Phe or 10^−6^ M phorbol 12-myristate 13-acetate (PMA), a protein kinase C (PKC) activator [41]. During the sustained phase of the contraction, FGAL (10^−8^ to 10^−3^ M) or the vehicle (distilled water + Cremophor^®^) was cumulatively added to obtain a concentration‑response curve [29]. The relaxation induced by FGAL was expressed as the reverse percentage of the initial contraction induced with both contractile agents, and the pD_2_ values of FGAL were calculated and compared.

#### 3.4.2. Effect of FGAL on Phenylephrine-Induced Cumulative Contractions

After the stabilization period, two consecutive and similar cumulative concentration-response curves for Phe (10^−10^ to 3 × 10^−5^ M) were obtained in the absence of FGAL (control). FGAL (3 × 10^−6^ to 10^−5^ and 3 × 10^−5^ M) was then added at different concentrations and preparations for 15 min, and a third cumulative concentration-response curve with Phe was obtained. Each preparation was exposed to only one FGAL concentration. The maximum amplitude of concentration-response curves for Phe was considered as 100% (control), and all contractions in the presence of FGAL were assessed referring to it. The pD_2_ value of Phe was calculated on the basis of the E_max_ reached in the presence of different concentrations of FGAL [42].

#### 3.4.3. Effect of FGAL on Rat Aorta Pre-Contracted with KCl (30 or 80 mM)

After the resting period, a contraction was evoked with 3 × 10^−7^ M Phe to test the organ responsiveness and maximum tension. Thirty minutes later, a second contraction was evoked with 30 or 80 mM KCl. During the sustained phase of the contraction, FGAL (10^−8^ to 10^−3^ M) was cumulatively added to obtain a relaxation curve. The relaxation induced by FGAL was expressed as the reverse percentage of the initial contraction elicited with 30 or 80 mM KCl, and the pD_2_ values of FGAL were calculated and compared [26]. 

#### 3.4.4. Effect of FGAL on Rat Aorta Pre-Contracted with Phe in Both Absence and Presence of K^+^ Channel Blockers

After the initial procedures, a contraction was evoked with 3 × 10^−7^ M Phe in both the absence (control) and presence of 10 mM TEA^+^, a non-selective K^+^ channel blocker [43]; 1 mM TEA^+^, a BK_Ca_ blocker [43]; 10^−5^ M glibenclamide, a K_ATP_ blocker [44]; 10^−4^ M BaCl_2_, a K_ir_ blocker [25]; 10^−3^ M 4-AP, a K_V_ blocker [45] and 5 × 10^−8^ M apamin, a SK_Ca_ blocker [46], in independent experiments, which were added to the organ baths 20 min before the Phe-induced contraction. During the sustained phase of the contraction, FGAL (10^−8^ to 3 × 10^−4^ M) was cumulatively added to obtain a relaxation curve. 

The relaxation induced by FGAL was expressed as the reverse percentage of the initial contraction induced with the agonist. The pD_2_ values of FGAL were calculated and compared.

#### 3.4.5. Effect of FGAL on CaCl_2_-Induced Cumulative Contractions in Depolarizing Medium (80 mM KCl) Nominally Ca^2+^-free

After the stabilization period, the Krebs solution was replaced by a Krebs depolarizing solution nominally Ca^2+^‑free for 45 min. After this period, two consecutive and similar cumulative concentration‑response curves with CaCl_2_ were obtained in the absence of FGAL (control). Then, FGAL (10^−5^, 3 × 10^−5^, 10^−4^ and 3 × 10^−4^ M) was added, at different concentrations and in different preparations, for 15 min, and a third cumulative concentration-response curve with CaCl_2_ (10^−7^ to 10^−1^ M) was obtained [47]. The E_max_ obtained with the control was taken as 100%, and all concentration‑response curves in the presence of FGAL were assessed referring to it. The pD_2_ value of CaCl_2_ was calculed based on the E_max_ reached in the presence of different concentrations of FGAL.

#### 3.4.6. Effect of FGAL on CaCl_2_-Induced Cumulative Contractions in the Presence of Verapamil and Phe

After the stabilization period, Krebs solution was replaced by a Krebs solution nominally Ca^2+^-free for 45 min. Next, 10^−6^ M verapamil was added to the organ bath for 10 min, followed by a contraction induced with 10^−6^ M Phe. Ten minutes later, two consecutive and similar cumulative concentration-response curves with CaCl_2_ (10^−5^ to 3 × 10^−1^ M) were obtained in the absence of FGAL (control). The preparations were then washed with Krebs solution nominally Ca^2+^-free, 10^−6^ M verapamil was added to the organ bath for 10 min, FGAL (3 × 10^−6^, 10^−5^, and 3 × 10^−5^ M) was incubated at different concentrations and in different preparations, and a third cumulative concentration‑response curve with CaCl_2_ was obtained [48]. The E_max_ obtained with the control was taken as 100%, and all concentration‑response curves to CaCl_2_ in the presence of FGAL were assessed referring to it. The pD_2_ value of CaCl_2_ was determined on the basis of the E_max_ obtained in the presence of different concentrations of FGAL.

#### 3.4.7. Effect of FGAL on Phe-Sensitive Ca^2+^ Mobilization from Sarcoplasmic Reticulum (SR)

After the stabilization period, Krebs solution was replaced by a Ca^2+^-free Krebs solution (with the addition of 3 mM EDTA) for 10 min, followed by phasic contractions with 10^−6^ M Phe until depleting the intracellular Ca^2+^ stores in SR. The bath solution was replaced by Krebs solution for 15 min to promote the replenishment of Ca^2+^ stores. The Ca^2+^-free Krebs solution was replaced for 10 min, and then two similar phasic contractions with 10^−6^ M Phe (control) were induced. FGAL (3 × 10^−6^, 10^−5^, 3 × 10^−5^ and 10^−4^ M) was incubated for 15 min at different concentrations and in different preparations, and another phasic contraction was evoked with 10^−6^ M Phe [28]. The inhibition of the phasic contractions induced with Phe was calculated by comparing the contractile response in both the absence and presence of FGAL.

#### 3.4.8. Effect of Phosphodiesterase (PDE) Inhibitors on Rat Aorta Pre-Contracted with Phe in Both Absence and Presence of FGAL

After the stabilization period, a contraction with 3 × 10^−7^ M Phe was evoked in both the absence (control) and presence of 3 × 10^−6^ M FGAL for 20 min [8]. During the sustained phase of the contraction, aminophylline (10^−10^ to 10^−3^ M), a non‑selective PDE inhibitor, milrinone (10^−8^ to 10^−4^ M), a PDE III inhibitor, and sildenafil (10^−8^ to 10^−4^ M), a PDE V inhibitor [38], were added to the organ bath, in different experiments, to obtain a relaxation curve. Their relaxation potency was evaluated by comparing their pD_2_ values in both the absence and presence of FGAL.

### 3.5. Statistical Analysis

Data were expressed as the mean and standard error of the mean (S.E.M.). The negative logarithm of FGAL, Phe or Ca^2+^ concentration that produced a half-maximal response (pD_2_, also denoted as pEC_50_) and the concentration of FGAL that inhibits 50% of maximal response to Phe or CaCl_2_ (IC_50_) were determined by non-linear regression [49,50]. Mean differences were statistically compared using Student’s *t*-test, for two independent groups, or one‑way ANOVA followed by Bonferroni’s or Dunnett’s post-test, for multiple comparisons. The null hypothesis was discarded when *p* < 0.05. All values were obtained using GraphPad Prism^®^ 5.01 software (GraphPad Software Inc., La Jolla, CA, USA).

## 4. Conclusions

**Figure 10:**
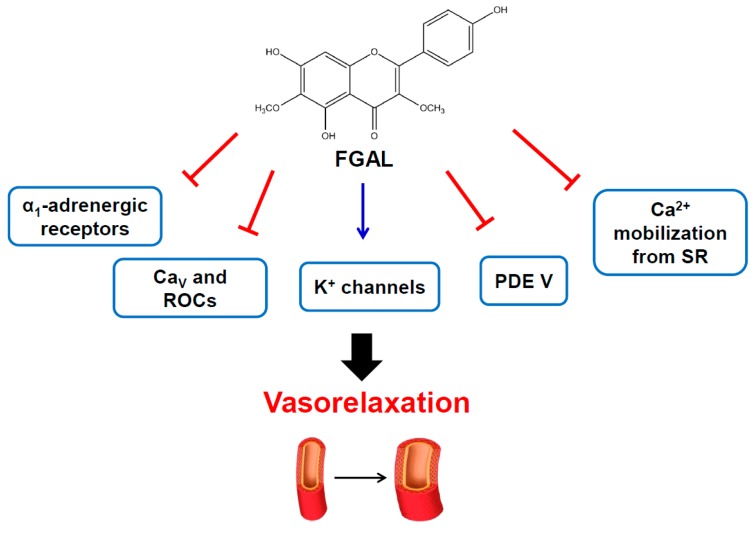
Overall study scheme representing the effect of FGAL on inducing rat aorta relaxation.

In this work, the mechanism underlying the vasorelaxant action of galetin 3,6-dimethyl ether in rat aorta was elucidated for the first time. This mechanism involves the noncompetitive antagonism of α_1_ adrenergic receptors and includes the non‑selective opening of K^+^ channels, the inhibition of Ca^2+^ influx through Ca_V_ or ROCs and the inhibition of intracellular Ca^2+^ release, which may be by direct or indirect mechanisms. Additionally, there is the involvement of cyclic nucleotide pathways, particularly through PDE V inhibition (Figure 10). Taken together, these data suggest that FGAL is a promising flavonoid to be used in the treatment of conditions associated with vascular smooth muscle disorders, such as hypertension or ischemia.
